# Contact Tracing Apps: Lessons Learned on Privacy, Autonomy, and the Need for Detailed and Thoughtful Implementation

**DOI:** 10.2196/27449

**Published:** 2021-07-19

**Authors:** Katie Hogan, Briana Macedo, Venkata Macha, Arko Barman, Xiaoqian Jiang

**Affiliations:** 1 Department of Bioengineering Rice University Houston, TX United States; 2 School of Engineering Princeton University Princeton, NJ United States; 3 School of Medicine University of Alabama at Birmingham Birmingham, AL United States; 4 Department of Electrical & Computer Engineering Rice University Houston, TX United States; 5 Data to Knowledge Lab Rice University Houston, TX United States; 6 School of Biomedical Informatics University of Texas Health Science Center at Houston Houston, TX United States

**Keywords:** contact tracing, COVID-19, privacy, smartphone apps, mobile phone apps, health information, electronic health, eHealth, pandemic, app, mobile health, mHealth

## Abstract

The global and national response to the COVID-19 pandemic has been inadequate due to a collective lack of preparation and a shortage of available tools for responding to a large-scale pandemic. By applying lessons learned to create better preventative methods and speedier interventions, the harm of a future pandemic may be dramatically reduced. One potential measure is the widespread use of contact tracing apps. While such apps were designed to combat the COVID-19 pandemic, the time scale in which these apps were deployed proved a significant barrier to efficacy. Many companies and governments sprinted to deploy contact tracing apps that were not properly vetted for performance, privacy, or security issues. The hasty development of incomplete contact tracing apps undermined public trust and negatively influenced perceptions of app efficacy. As a result, many of these apps had poor voluntary public uptake, which greatly decreased the apps’ efficacy. Now, with lessons learned from this pandemic, groups can better design and test apps in preparation for the future. In this viewpoint, we outline common strategies employed for contact tracing apps, detail the successes and shortcomings of several prominent apps, and describe lessons learned that may be used to shape effective contact tracing apps for the present and future. Future app designers can keep these lessons in mind to create a version that is suitable for their local culture, especially with regard to local attitudes toward privacy-utility tradeoffs during public health crises.

## Introduction

At the end of 2019, a novel coronavirus was determined to be associated with a group of pneumonia cases in Wuhan, China. In 2020, this novel coronavirus spread rapidly throughout China and the globe, prompting the World Health Organization to name the disease COVID-19 and the virus associated with the disease SARS-CoV-2 [1]. By October 2020, the COVID-19 pandemic had resulted in over 200,000 deaths in the United States [2] and over 1,000,000 deaths globally [3].

Strategies to monitor and control the spread of COVID-19 have hinged around a combination of traditional and nontraditional strategies, including rapid testing, self-quarantine, and contact tracing. Contact tracing has formed a key component of the plans to control the spread of infectious diseases in recent years, yielding a wealth of literature concerning its importance and efficacy. Contact tracing traditionally involves interviewing infected individuals and following up with any close contacts to communicate increased risk due to exposure and provide information and strategies related to that risk [1]. Alongside face-to-face contact tracing, various digital strategies have been employed to mediate this process and increase efficacy, including the introduction of a wide variety of contact tracing apps. In an article modeling the efficacy of contact tracing for Ebola, Browne et al [4] identified and examined key epidemiological parameters that impact the efficacy of contact tracing, including incubation period, infectious period, and monitoring protocols. During recent Ebola outbreaks, contact tracing apps developed in Guinea and Sierra Leone have provided a means for contact tracers to increase the speed and accuracy of contact tracing and efficient centralization of real-time data, as well as the coordination of resources and interventions [5,6]. These studies suggest that technology may play a key role in increasing the efficacy and timeliness of contact tracing. In contrast to the studies above, which placed technology in the hands of contact tracers, most strategies for COVID-19 contact tracing implementations use available smartphone technology to actively monitor risk for users in the general population.

A study by Ferretti et al [7] modeled the use of contact tracing apps and concluded that widespread use of these apps could be used alongside strategies such as widespread testing and physical distancing to suppress the pandemic successfully. This model suggests that contact tracing apps could allow greater freedom of movement, but low app adoption or incorrect usage could lead to continued spread. Similarly, models developed by Yasaka et al [8] determined that although some disease suppression could be seen with 25% adoption of their app, higher population uptake (75%) would be required for more substantial reductions of the infection curve. A primary concern, therefore, in determining the potential efficacy of a contact tracing app is the number of people using the app and the efficiency and accuracy of its information distribution. Rowe [9] outlined three conditions necessary for the success of a contact tracing app: (1) correctness of information including diagnosis, (2) high likelihood of smartphone presence during contact, and (3) a high proportion of people using the app. As a result of the myriad technological changes that have occurred in recent years, utilizing digital technology to perform contact tracing is both a truly promising solution and an unprecedented problem. Therefore, extracting lessons from the current pandemic and the role technology can play will serve as crucial components to optimizing public health strategies during current circumstances and future outbreaks.

There has been a wide variety of COVID-19 contact tracing app reviews published over the course of the pandemic across a range of subjects, including technical analysis [10,11], privacy concerns [12-14], and ethics [15-18], each of which present valuable information on a specific aspect of digital contact tracing. This viewpoint is designed to combine information from across the expanding COVID-19 digital contact tracing literature and address key considerations that must be taken into account for developing and refining future contact tracing app design. This paper aims to accomplish this goal by providing background on common strategies for app-based contact tracing, discussing the advantages and limitations of several prominent COVID-19 contact tracing apps, and elucidating the privacy and nonprivacy concerns that have affected their adoption and reliability during the COVID-19 pandemic. Table 1 summarizes the key points of each section. This paper is intended as a hermeneutic literature review, with analysis that provides a specific viewpoint on the subject at hand. As such, a literature review was conducted over the course of July 2020 to March 2021 via PubMed and Google Scholar using terms including COVID-19, contact tracing app, and digital contact tracing, etc.

**Table 1:** Key considerations for the design of contact tracing apps, including those that relate to structure, technology, adoption requirements, and privacy.

App characteristic	Considerations
App structure	Centralized Generated user data stored or managed in a central server Direct oversight of user data Decentralized Generated user data stored or managed on user devices User privacy and security advantages
App technology	GPS and location-based tracking Additional potential for privacy concerns Higher noise compared to Bluetooth May be paired with proximity-based tracing for increased accuracy Bluetooth and proximity-based tracing Fewer privacy concerns, particularly when paired with a decentralized structure High noise—signal attenuation and accuracy issues due to environmental signal absorption and reflection
Adoption requirements	Mandatory May be viewed as a privacy violation Higher adoption rates may increase the accuracy and efficacy of the app Opt-in Voluntary use with specific permissions to address privacy concerns May have lower adoption rates that decrease the accuracy and efficacy of the app
Privacy	An app should offer privacy from other users, the app manager, and snoopers The privacy-utility tradeoff of an app must be shaped around the local cultural attitudes Privacy and security have been repeatedly stated as primary concerns for app users: successful apps should adopt a privacy-by-design structure
Other	An app should be easy to use and reduce user fatigue Battery drainage, interference with other medical apps, and incompatibility with some phone models have proved to be barriers for successful global deployment of some contact tracing apps Users must be encouraged to follow all other precautions to limit the spread of disease (ie, recommendations from public health authorities like mask wearing and physical distancing)

## Key Considerations in App Design and Categories

In this section, we outline some of the common considerations for developing contact tracing apps, namely strategies and technologies employed. We discuss the advantages and disadvantages of each of these strategies, particularly with regard to efficacy and frequency of use of contact tracing apps globally.

### App Parameterization Using Epidemiological Data or Disease and User Characteristics

When developing new apps for contact tracing, several factors must be accounted for within algorithms and interfaces to ensure accurate information and notifications for all parties involved. For instance, rates and types of transmission must be incorporated into the app design in order to determine the number and time frame of contacts that must be notified and tracked. The basic reproduction number (R_0_) accounts for baseline disease transmissibility without immunity from exposure or vaccination or any intervention to prevent transmission [19]. Transmission for COVID-19 falls into the categories of symptomatic, presymptomatic, asymptomatic, and environmental transmission [7]. Contact tracing generally most accurately accounts for symptomatic and presymptomatic transmission, as asymptomatic and environmental transmission may not be readily identifiable. However, when contact tracing is paired with large-scale community testing, there is an enhanced ability to model transmissibility, accounting more accurately for asymptomatic and environmental transmission.

Incubation and infection times must also be taken into account to narrow time windows over which contacts should be identified for potential exposure. For COVID-19, the average time to symptom development is 5.1 days, with 99% of symptomatic cases displaying symptoms by 14 days [20]. In a study of infectiousness profiles for COVID-19 infector-infectee transmission pairs, the highest viral load was observed at symptom onset, translating to increased transmission risk [21]. However, 44% of secondary cases developed as a result of exposure during the index patient’s presymptomatic stage, allowing COVID-19 to spread rapidly and highlighting a heightened risk when the perceived threat is low. Indeed, the disease’s highest transmissibility has been reported to be before or immediately after symptom development [22]. Therefore, when modeling the timescale over which a patient may have been infectious, contact tracing apps must account for possible transmission up to 2 weeks before symptom development or a positive test result for asymptomatic carriers. With the incorporated passive observation of individual location or contacts, this strategy may help to account and partially compensate for diagnosis delay, which was observed to lead to increased spread, particularly as communities developed and launched containment strategies early in the pandemic [23]. These parameters are essential in initial app development and determining individual risk but should also be considered when determining the amount of time necessary for data storage, particularly given the privacy concerns voiced over the long-term collection of location and health data [14].

Additionally, some thought should be given to the direction of contact tracing with the collected data. Forward tracing, used almost exclusively within current contact tracing apps, works forward from a current positive diagnosis to identify the contacts, particularly during peak infectiousness, who are now at risk of contracting the disease [24]. Backward tracing, however, works back from the current diagnosis to identify the secondary origin of transmission and find additional contacts who are now at risk of having the disease [24,25]. This method of tracing seeks to find the source of an outbreak and has been successful at identifying clusters around an individual known to be contagious. For instance, backward tracing was used to identify clusters and prevent further community spread of COVID-19 in both Japan and Singapore early in the pandemic [24,26]. Likewise, combining these techniques in bidirectional tracing has been shown to be particularly important with a long incubation period, with models showing a 2-fold reduction in effective reproduction number versus forward contact tracing alone [24]. Currently, most contact tracing apps exclusively use forward contact tracing, which fails to take advantage of the full range of data available. Digital tracing allows for easier extension of the tracing window in a bidirectional manner because there is no need to rely on patients’ memories. However, if app usage is not high enough within a local population, digital tracing in either direction may be disrupted by network fragmentation and insufficient data [24]. Therefore, in addition to the epidemiological data necessary for app parameterization, due diligence must be given to increasing app usage numbers to increase the efficiency of a particular strategy, even those shown successful in manual tracing and modeling.

When developing app parameterization and settling on technological strategies and techniques for implementation such as those discussed below, app developers must ultimately choose strategies that result in the highest efficacy and accuracy. This strategizing must consider in particular app false positive and false negative rates for the app. False positives (type I error), referring to users incorrectly notified of increased exposure risk, can put undue strain on health care infrastructure by increasing demand for testing, result in increased levels of highly negatively impactful enforced quarantine, and decrease app utility by decreasing user attentiveness to app notifications [10,27]. False negatives (type II error), conversely, refer to individuals who have had close contact and are at high risk but are not identified by the contact tracing app, which may be the result of low app sensitivity or improper tracing [10]. These high-risk individuals are ignorant of their risk and may contract the disease and spread it further in the community. The best policies and parameters based on this reasoning seek to minimize false negatives first, as these will result in further untracked community spread. These strategies, however, will likely result in a high number of false positives due to prioritization of high-sensitivity, low-specificity methods [27-29].

False positives may result through a variety of means, but initial planning to prevent false positives must begin with the technical strategy chosen and the policy-based design of initial parameters. The definition of close contact (6 feet or 2 meters, 15 minutes) most commonly put forward by public health entities has been argued to be too coarse for mass tracing, as evidenced by the high number of false positives seen with manual contact tracing and that this definition has resulted in decreased accuracy with digital contact tracing as well [27,28]. Likewise, as will be discussed further below, the technical strategies employed, such as GPS or Bluetooth, will directly impact the accuracy and efficacy of an app [11,30]. Signal strength and duration selections for the app, as well as firmware and software compatibility with the app and other users’ devices, will play a role [27]. These considerations make it imperative that apps undergo significant real-world testing to determine efficacy, data from which has not yet been revealed for most available COVID-19 contact tracing apps.

### Centralized Versus Decentralized Architecture

The decision to utilize a centralized, decentralized, or hybrid overall structure or strategy is a key initial consideration when designing and implementing contact tracing apps and requires balancing privacy, security, and efficacy concerns. Centralized apps use strategies that employ a main server for data storage and analysis. Conversely, decentralized apps feature data storage that is distributed across the user network, with no individual entity having complete control or information access [31]. A hybrid architecture may have a component of both approaches, with some information handled on individual devices with a central server analyzing data and sending notifications. Contact tracing apps using each of these architectures have been employed for COVID-19, with the choice of structure highly dependent on government and cultural norms in the region of use and the needs of public health officials.

A centralized contact tracing app architecture may require significant trust in the beneficence of government investment and national or regional data infrastructure. In a centralized approach employing technology like Bluetooth LE, for example, a trusted third party (TTP) such as a government or public health entity may assign users an encrypted identifier that is broadcasted during app use [32]. These encrypted identifiers are broadcast to other users, with apps storing identifier lists that may be sent to the main TTP server in the event of a positive diagnosis. Then, users who appear on this list will receive notification of risk from the TTP through their app. Some notable examples of COVID-19 contact tracing apps with a centralized architecture include Singapore’s TraceTogether and China’s Health Code apps [33,34].

Conversely, decentralized architectures remove the role of data accumulation and analysis from a central server and instead place these functionalities on user devices. Anonymous user identifiers are generated as random seeds with a short lifetime (chirps) that are exchanged with other user devices. Upon a positive diagnosis, a user may upload seeds and accompanying temporal data to a central server. By analyzing seeds and temporal data from positive users on the central server, exposure notifications are generated on user devices. In this way, the central server does not serve as the primary driver of risk assessment, nor, due to the anonymized or pseudoanonymized nature of seeds and chirps, can identifying details be derived from information available on the central server [10]. There has been a significant shift in interest toward decentralized or hybrid app architectures in recent years for privacy and security reasons, with a model focused on location privacy also indicating lower data retrieval times compared to centralized architectures [35]. Centralized approaches may be at risk of a variety of privacy and security attacks, including deanonymization of personal data through direct breaches of the main server. Likewise, decentralized app structures may also yield privacy and security concerns. For example, while Bluetooth LE and other technologies that have been applied in decentralized structures may record proximity-based data instead of positional data, personal medical information may be extrapolated via a linkage attack that uses data concerning specific, close contact notifications and known positional information from other devices to determine disease status, as explored by Bengio et al [12] in an excellent recent review of security concerns for decentralized app designs. Therefore, the decision to utilize a centralized versus decentralized app strategy centers on tradeoffs between culturally specific privacy concerns and structural trust needed for high population uptake as well as security concerns.

Additional security measures can and should be developed for both centralized and decentralized structures. Alongside encryption and anonymization techniques, blockchain technology has been suggested as a tool for increasing security and data privacy, particularly for decentralized strategies. Blockchain technologies seek to decentralize data by duplicating and distributing information across a global computer systems network, making it difficult to spoof or manipulate data within these distributed databases [36]. Xu et al [37] have developed a blockchain method called BeepTrace, designed as a bridge between users and central servers that reduces the vulnerability of sensitive data such as user identification and location, with the ability to predetermine and regulate the length of data storage. This proposed blockchain would also allow for public accountability of governments and corporations via transparency and ease of information verification, as in the case of manipulated efficacy data. However, in practical implementation, strategies would require refinement to reduce the intensive computational requirement for large populations, particularly dense populations such as in India and China.

### Location- and Proximity-Based Tracking

#### GPS

GPS and global information systems (GIS) are routinely used for large-scale disease monitoring and predicting disease spread, as seen with features such as Google Flu Trends, which have been used to predict the spread of seasonal flu in the United States [38]. Similarly, for COVID-19, online macroscale tracking systems were developed early in the pandemic to allow for up-to-date information on the global and regional spread of the virus. The timely updates of these online GIS and mapping dashboards, including the Johns Hopkins University Center for Systems Science and Engineering dashboard and Who Health Organization Dashboard, provided a means of data sharing concerning outbreak events for the public [39].

Alongside macroscale information, GPS and GIS technology may be employed for individual contact tracing via GPS and social media mapping. The ubiquitous use of GPS-enabled smartphones in many regions of the world provides an opportunity to collect spatiotemporal trajectory data for individuals. One example of this is the STRONG (Spatiotemporal Reporting Over Network and GPS) strategy, proposed and published by Wang et al [40], which analyzed the backend GPS spatiotemporal data collected through the social media app WeChat to trace the close contacts of users, primarily in China. This strategy offers a means of integrating cell phone–based GPS positioning with voluntary real-world transaction data that provides accurate timestamps and position information, although this also requires a substantial relinquishment of individual privacy. Similar to this strategy and adopted soon after the STRONG system was published, the Chinese government began employing a national monitoring system, which, as opposed to GPS data, uses cell phone base station positioning data to evaluate individual exposure and risk [40,41]. Other countries had also employed government-sponsored cell phone location data collection to track individuals and prevent COVID-19 spread, including South Korea, which notified users before entry into “high-risk” zones, and Israel, which used location data to inform contacts of confirmed infected individuals and for quarantine enforcement [42]. Additional apps developed by countries that involve location-based tracking include the Rilevatore Teramoto app released by Italy and apps from Austria (NOVID20), India (Aarogya Setu), Norway (Institute of Public Health app), and Spain (Open Coronavirus), which combine location and proximity-based tracing [32].

Therefore, GPS and location-based mapping have been widely viewed as a potential tool within the context of COVID-19 contact tracing apps. However, further refinement is needed to reduce “noise” within GPS-based systems, which may reduce the efficacy of the system to identify high-risk contacts or areas with sufficient specificity [40]. The granularity of location-based GPS data may not be sufficient to determine a distance of 6 feet (2 meters), with GPS positioning error at approximately 10 meters indoors [11]. This limitation may lead to significantly increased false-positive and false-negative rates, and hence reduce the accuracy and efficacy for GPS-based contact tracing strategies. Bluetooth proximity-based systems are generally considered to be more accurate, with fewer false positives reported [32]. Additionally, privacy concerns arise with the thought of highly specific spatiotemporal tracing, particularly when combined with timestamped real-world interactional data available through social media networks, which may generate problems with adoption. Security concerns must also be considered, as GPS systems are particularly vulnerable to spoofing attacks, in which a fabricated GPS trail may send incorrect spatiotemporal data to a receiver [43].

One example of a privacy-forward, GIS-based app is the COVI Canada app, initially proposed for government use, although the Canadian government has moved in another direction [44]. The proposed app employs GeoIP services to yield coarse location data that maps risk-stratified zones through which users have moved. Additionally, Bluetooth-based contacts are recorded among individuals. COVI Canada claims a decentralized approach in which pseudonymized personal data such as the number of contacts and diagnosis status are encrypted and sent to the main server where risk is calculated for each user daily via artificial intelligence (AI). The coarse location data yielded by this approach may also provide heat maps of outbreaks to public health authorities.

#### Bluetooth

In contrast to GPS mapping, Bluetooth records interactions between individuals based on device proximity. When individuals are in close proximity, Bluetooth-based token sharing allows for a precise record of the interaction. In this case, the limited range of Bluetooth-based technologies becomes advantageous for recording only close-range interactions to approximate a 6-foot distance. Additionally, the relative signals’ strength can be used to determine the approximate distance between individuals, allowing for proximity tracing of individuals in high-risk settings, such as indoor environments or public transportation [32,45]. However, this strategy requires high adoption for accurate proximity tracking and risk assessment, as it requires a direct interaction between users. Bluetooth has an approximately 10-meter location granularity, with visibility between devices possible up to 30 meters apart [11]. Signal attenuation may be used to indirectly assess distances between devices, although this is not linear. Additionally, high standard deviation in received signal strength between 2 and 6 meters decreases accuracy within this range, and with 2 meters used to indicate close contact, it may lead to decreased app efficacy and increased false positives using Bluetooth technology.

Additionally, although Bluetooth-based tracing is generally considered more accurate than GPS-based strategies, significant issues are present with signal attenuation and accuracy. Signal absorption and reflection by the surrounding environment can lead to inaccuracies in reported distances between users [46,47]. In a recent paper on this topic, Leith and Farrell [48] evaluated the efficacy of Bluetooth LE technology for COVID-19 contact tracing in real-world environments, including users walking in the city, at a meeting table, in a train carriage, and grocery shopping, as well as assessing the impact of device orientation, use of a handbag, and type of indoor wall on signal attenuation. After observing significant impacts from all of these factors and no corresponding decrease in signal strength with increasing distance, the authors called for extensive real-world testing of Bluetooth-based COVID-19 contact tracing apps as well as data to inform the efficacy of such apps compared to manual contact tracing.

Bluetooth-based strategies have become common, with some notable examples, including national apps in several countries. Singapore’s TraceTogether app was the first such Bluetooth-based, government-sponsored contact tracing app [33], and since then, the list has grown to include the Pan-European Privacy-Preserving Proximity Tracing (PEPP-PT) app, released by a European consortium, as well as Australia’s COVIDSafe [49] and Canada’s ABTraceTogether [44]. In contrast, the industry-based joint Apple-Google contact tracing app also relies on Bluetooth proximity tracing, but concerns have arisen around private companies’ handling of sensitive health data and this new role for large tech corporations in the public health arena [50].

The PEPP-PT app was initially touted by its creators as having a 90% true-positive and 10% false-negative rate [51]. However, several studies since have indicated that Bluetooth-based proximity tracing may have significantly high error rates. For instance, in a study by Girolami et al [52] involving high school students, a traced interaction accuracy of 81% was obtained. However, in initial planning, 42% of student devices were found to be incompatible with active Bluetooth beaconing and unusable for contact tracing, which would pose a significant issue in the population at large. In a real-world setting, devices will have different versions of Bluetooth technologies and may receive, transmit, and damp signals at different levels. Likewise, at least 50% beacon loss between device dyads was observed, posited to be due to device positioning and unpredictable environmental signal attenuation as discussed above. This study, alongside real-world testing of the Bluetooth-based Google/Apple Exposure Network (GAEN) by Leif et al [48], points to somewhat unreliable and unpredictable efficacy and utility for Bluetooth-based apps and demonstrates the need for further innovation and design.

#### Contact Points

One alternative to location-based GPS or Bluetooth tracking, which allows for more user privacy, uses an opt-in system of contact points. Yasaka et al [8] proposed the idea of an app that allows users to host or join checkpoints at which other users may also check in by scanning a generated location quick response (QR) code. Ideally, users would generate check-in QR codes for any gathering or public place, which poses a risk for COVID-19 transmission, and when a user tests positive, all users who have checked in at locations with them over the potential infectious period will receive an updated risk level. High user adoption would be necessary for such a system to be efficacious. Additionally, apps that rely on sustained conscious use pose a problem with user fatigue, with drop-offs in check-ins providing the potential for inaccurate risk assessments.

### Volunteered Health Status

An alternative to tracking or tracing apps is an app that allows for volunteered health or contact information to be entered to initiate case-based contact tracing outside of the app itself. This tactic of voluntary information entry mitigates some privacy concerns centered around tracking and tracing. The two main categories of apps falling into this category are symptom monitoring apps and case-initiated notification systems. These apps do not directly perform contact tracing but instead serve to use technology to simplify the contact-tracing process.

Symptom monitoring apps rely upon users to accurately record a log of personal recent health records to assess for COVID-19 risk based on symptomology and may be used most effectively as a screening tool for identifying symptomatic cases. These apps require regular logging of recent symptoms associated with COVID-19, including fever, dry cough, shortness of breath, fatigue, muscle aches, and loss of taste or smell. Yamamoto et al [53] reported an example of this system integrated into an already established health screening app, K-note, which significantly decreased the follow-up burden on health care providers for monitoring close contacts of COVID-19–positive cases, allowing for efficient algorithmic identification of symptomatic users. Additional personal data that might be logged includes COVID-19–positive contacts, travel history, and visits to health care institutions, allowing for further risk stratification. Another such app includes the one implemented by Yap et al [54], COVID-19 Symptom Monitoring and Contact Tracking Record (CoV-SCR), which provides a designated space for users to track symptoms (rated 1-5) as well as keep a 14-day record of travel and close contacts whom the user may notify in the event of a positive diagnosis. Medical and academic institutions have already employed such efforts to keep a regular log of potential symptoms as a means of preventing spread, providing a way to do a first-pass screening of individuals and identify those who may need to be tested or isolated. Additionally, symptom monitoring may be paired with in-person screening, such as temperature checks upon entry to an institution. While many such apps are institution-specific, such symptom logging may be integrated into currently available personal health record apps.

Early identification of potentially COVID-19–positive individuals may enable efficient tracing of contacts and early isolation. However, such apps rely upon the memory and honesty of users to accurately portray health status. Additionally, this strategy fails to account for asymptomatic individuals, who may slip through the holes in this system and further spread the virus. Users may also be unable to account for strangers or individuals encountered in public settings such as public transportation. Passive tracing and tracking apps have therefore become the preferred avenue of exploration and implementation for apps intended for large-scale use, as discussed in the examples below, most of which seek to combine symptom monitoring with location- or proximity-based tracing.

### Mandatory Versus Voluntary Use

There are three potential strategies for enforcing such an app at a federal level: opt-in, opt-out, or mandatory use. An opt-in model allows people to download the app if they so choose and has been advocated for on the basis of consent for acquisition, use, and sharing of personal information [23]. An opt-out model would automatically provide the app for all, but users would have the option to delete the app if they preferred not to use it. The final model is a mandatory download of the app for all people without deleting it. Mandatory app usage may be enforced by preventing people from using public services or entering buildings if they do not have the app. China, Hong Kong, Taiwan, and South Korea have used the mandatory model [55]. Many ethical concerns have been raised about such a design, including rights to personal autonomy and informed consent [16]. However, another concern is widening disparities between those who have access to relevant technology such as the internet and Bluetooth-capable smartphones and those who do not. In addition to an inability to move freely or engage meaningfully in public life, those who do not have smartphones or new operating systems do not have access to incentives to increase app use, such as those Parker et al [18] suggest like funds for charity donations and free mobile phone credits. Overall, the ability of a country to enforce a mandatory download system will depend on the socioeconomic status and cultural values of its citizens, with some nations more likely to experience significant pushback against or widespread inefficiencies in a mandatory system.

Furthermore, once the app is downloaded, behavior on the app may be mandatory/opt-in/opt-out. For example, once the app is downloaded, even if the app itself is optional, location sharing may be nonvoluntary and continuous. Other apps may allow for opt-in for location sharing. Although this may minimize the app’s efficacy, this approach allows for greater user privacy if they are venturing somewhere that is private to them. Additionally, apps may require mandatory sharing of infection status, as is the case in Singapore.

## Specific App Examples of Successes and Limitations

In this section, we outline some of the large-scale implementations of contact tracing apps, most notably those created by national governments. We will discuss the successes and downfalls of some of these implementations and touch on how local cultural attitudes influenced the design of each app and how it was received by the local population.

### Singapore

Singapore’s TraceTogether app was the first national deployment of a Bluetooth-based contact tracing system in the world. The app was presented as being “for the people,” with the ultimate goal of protecting the population [33]. This Singaporean technology provides several lessons for contact tracing, including concerns about Bluetooth, the privacy-utility tradeoff, as well as centralized and decentralized systems. Most importantly, the Singaporean contact tracing strategy reveals how cultural attitudes and norms must be taken into account within a community in order to achieve maximal success.

Aimed at transparency and international cooperation, the Government Technology Agency of Singapore published information about BlueTrace, the protocol that underpins TraceTogether as well as OpenTrace, an open-source repository for other countries that heavily influenced Australia’s national app design [56]. The app features a hybrid decentralized-centralized, proximity-based approach and functions through contacts exchanging non–personally identifiable messages, with frequently rotated identifiers for security and privacy. Encounter history is kept on local storage and thus is decentralized. While the app itself is not mandatory, once a user is tested positive, they are legally required to release their stored data to the government per the Infectious Disease Act [55], after which health officials reach out to contacts based on personal identifiers. At this point, a user’s data is stored on a centralized government server [57], and the government retains the right to share this information with other groups, including other governments [55,58]. It should be noted that these requirements would not be the same across countries and communities. The government generally advertised the TraceTogether app as being a privacy-by-design system because of the decentralized setup [59]. However, this is only true for healthy individuals who do not have to share their data to the centralized server for government control.

The BlueTrace authors also note other privacy-by-design implementation choices for the app. The TraceTogether app claims to keep proximity data only for 21 days, therefore limiting the amount of unnecessary data stored [56]. Furthermore, the BlueTrace report claims that users have control over their data, and all of their information is deleted upon request [56,59]. The TraceTogether system requires a health official to confirm that a case is legitimate to avoid false self-reported positives that may stir up panic and decrease the program’s legitimacy and trust. However, the additional step of health official approval may be difficult to implement in other nations with high rates of uninsured citizens, who may not be able to seek medical care. This concern will inevitably affect some countries more than others, notably based on the presence or absence of universal health care.

Since the system requires interaction between two users, an increased percentage of people who download the app increases the program’s effectiveness quadratically [56]. While Singapore was reaching relatively high usage as compared to other opt-in/opt-out programs, the 17% download rate was still insufficient to reach maximal efficiency [60]. However, the government hesitated to make the app mandatory to prevent pushback regarding surveillance and control concerns. There were some concerns that the Singaporean government was collecting cell phone data through the app, although the government denied these claims [61].

The app itself had several issues in terms of functionality. For example, certain types of phones, including Apple products or older models, could not download the app or experienced severely limited functionality. Further, there were lags between user contacts and device communication and logging. There are also inherent limitations to Bluetooth, including material barriers that attenuate transmission signals. Additionally, Bluetooth technology can interfere with other health-related apps and implantable devices [62]. High variance in transmission power across device types may also lead to difficulty in assigning appropriate thresholds to determine close contact distances relative to signal strength. Battery usage issues have also been reported [56].

To address some of these technical issues and privacy concerns, the government eventually pivoted to distributing a wearable device to all of its citizens, which would complement the TraceTogether app. These devices would not contain any information beyond contact history, thereby protecting mobile cell phone data. Further, since the devices were identical, they would bypass some of the aforementioned technical issues across phone models and provide access to all. However, this implementation did not reduce concerns about location surveillance. In contrast to GPS, which tracks location, Bluetooth tracks interactions, which means that interactions between lawyers, doctors, and journalists are no longer confidential to the government [55]. Further, even with Bluetooth, it is still possible to narrow down one’s location, especially as data accumulates [57].

Chua et al [61] notes that America may be unable to mimic Singapore’s system because of cultural attitudes toward privacy-health tradeoffs. This is generally true across all distinct countries, which will each require individual policies that fit their population. The app developers themselves note that this system is specific to Singapore [58].

### Apple/Google

On April 10, 2020, Apple and Google announced a joint effort to create a contact tracing app framework that would serve to facilitate cross-platform monitoring as public health officials globally developed apps for their respective jurisdictions [59]. Phase 1 of this effort focused on releasing an application programming interface (API) for interoperability between iOS and Android systems, which was released to public health officials in May. This release was accompanied by signs that these corporations had reached out to large nations to create some patches between their initial health app designs and the GAEN API, notably Australia [59]. Following this, phase 2 released a means of building access to public health apps into the iOS and Android platforms through an Exposure Notification app provided with software updates. This system allows for Android and iOS users to opt in to an Apple and Google initiative that also ties in local contact tracing apps [59].

The API functions allow Android and iOS devices to exchange data with each other for improved contact tracing. Additionally, this framework features an opt-in, decentralized contact tracing system that relies on Bluetooth technology. The main emphasized advantage of the GAEN API was initially privacy, with no mechanism for recording users’ location data. A new Temporary Exposure Key is generated once daily, with Rolling Proximity Identifiers (RPI) generated every 10 minutes. Beacons with RPIs are broadcast every 250 ms, while devices scan for beacons every 4 minutes. The signal strength of received beacons is used to determine distances between users, while the number of beacons may determine the duration of contact exchanged [45,50]. Close contact is generally defined as 15 minutes of contact at distances of less than 2 meters. The exact parameters that qualify a signal as a significant contact may be determined by public health officials, with variable attenuation thresholds chosen by the nation [45]. GAEN API–based apps have been utilized worldwide, particularly within Europe (eg, Germany, Switzerland, and Italy).

The value of Bluetooth-based apps is a precise record with contacts who may be excluded or difficult to trace with traditional contact tracing methods, such as strangers in public settings. However, GAEN API–based apps have recently come under scrutiny for issues with efficacy in these scenarios. Leith and Farrell’s [45] study sought to apply the GAEN API in a real-world public transportation setting, recording measurements on a commuter train between handsets at greater and less than 2 meters for 15 minutes. Using Swiss and German attenuation thresholds from apps released in late May and early June, no close contacts were recorded. The Italian attenuation threshold yielded 50% true positives with a 50% false-positive rate. While this study only used Android devices, it yielded data that suggests significant changes in noise levels based on individual use. Additionally, signal strength was demonstrated to have complex interactions with the environment in which measurements took place, including signal reflection by tram walls and absorption by bodies (at 2.4 Hz), with no clear trend in signal attenuation with respect to distance based on these complex effects. Although more such studies are required, this data suggests that the inherent flaws of Bluetooth-based contact tracing, with small changes such as models of devices and signal absorption and reflection having an outsized impact on outcomes, may significantly decrease the efficacy of the GAEN API. This limitation may not be unique to the GAEN API and is indeed of concern for all Bluetooth-based contact tracing apps.

Although this initiative was initially well received, in addition to issues of efficacy, several concerns surrounding the imposition of large tech corporations’ influence in the spheres of public health and politics have arisen. These doubts have centered around a questionable past for both corporations regarding data management as well as the potential for mission creep for large tech companies [50]. The increasing inroads of tech corporations into biomedical fields, as in developing AI medical diagnostics, electronic medical records systems, and software kits for clinical trials, have some worried that in the rush to develop contact tracing apps, traditional medical expertise and professional knowledge has been traded for efficiency and optimization, which are the key values of technological production [50]. In addition to this, upon release of the GAEN API, Apple and Google have refused to work with nations and public health entities with apps in development that feature a centralized approach. This strategy effectively undermined government attempts at app creation in France and Latvia by refusing technical expertise [50]. Agencies have been forced to create workarounds for their apps that are unstable and battery-draining [59]. This lack of good-faith effort in these situations has been seen as evidence that public health and privacy concerns are currently being used as a means of increasing market share through contact tracing app development.

### Ireland

Ireland’s COVID Tracker app has been highlighted as a successful adaptation of the GAEN API for national use. As such, the app functions as a decentralized, Bluetooth-based system aimed at preserving individual privacy [62,63]. One of the most significant accomplishments of the opt-in app was its relatively rapid adoption, with 37% of the population having downloaded it within a week of release [62,63]. Over the course of July and early August, 137 users had been notified of their potential exposure to COVID-19 [62]. The success of the Irish app has led to its government working with other nations to retool the app for their use, including the United States [62].

Prior to launch, Irish health officials requested feedback from academic researchers and data and visualization experts as well as civil societies to evaluate the app, which has been noted as a potential source for public trust and perceived efficacy [64,65]. According to the prerelease report card issued by the Irish Council for Civil Liberties and Digital Rights Ireland, however, there were concerns regarding app privacy and security structure [64]. Most significantly, these groups noted the lack of efficacy data to back up claims of high accuracy, the need for timely deletion of personal data which could be extrapolated to yield user location, and concern over the use of closed-source Apple/Google software and control of health data by foreign private entities.

### China

The Chinese tech group Alibaba released their Alipay contact tracing app Health Code on February 9 in Hangzhou [33,66]. Soon after, Tencent released a similar system on WeChat. The QR code-based Health Code app has a broad audience in China, with the app used in over 300 Chinese cities with at least 900 million users as of August 2020 [33]. Through public-private partnerships and centralized government monitoring, Health Code has now become mandatory for access to public spaces in many areas. The app uses a color-based QR code system that reveals user risk—green, yellow, or red. Those with a green QR code may visit public spaces and others, while those with a yellow or red QR code are subject to self-isolation for 7 to 14 days [33,67]. App data pairs actively collected data such as self-reported symptoms, address, and government ID with passively collected GPS location data, online transaction data, and surveillance via facial recognition technology, CCTV (closed-circuit television), and drones, all of which are monitored and synthesized by a central government server to determine exposure risk and generate the QR codes [66-68]. The users’ status is updated daily at midnight to account for the previous day’s activities [33].

The mandatory acceptance of this app has been cited for enabling greater effectiveness versus voluntary acceptance for reducing the spread of the virus. Additionally, the app’s widespread use has demonstrated a much stronger tracing record versus South Korea, which only requires diagnosed individuals or close contacts to download their app [67]. However, the mandatory, centralized structure of this app may be responsible for considerable user stress. In one study by Joo and Shin [67], users reported that issues with app inaccuracy and errors were less stressful overall than privacy concerns. Because public spaces are restricted, even without a mandate, a de facto mandatory environment would exist for users who want to participate in public life, such as going to work or seeing family members, and could exclude those without access to appropriate technology or reliable internet access, highlighting the complexities of regulating movement based on exposure risk [66].

Likewise, significant concerns have been raised regarding erroneously issued yellow and red QR codes due to incorrect data entry or technical errors, which may necessitate unwarranted self-isolation [67]. Conversely, false-negative green codes have been reported in Wuhan, with COVID-19–positive individuals given a pass to public areas [33]. Users have reported difficulties in having an incorrect code corrected within the centralized system, adding to technological stress associated with the app [67]. Inconsistencies at the local level have added confusion to this process as users move between regions, with a yellow code requiring 7 days of quarantine in Hangzhou versus 14 days in Shandong [33]. These issues have been paired with a lack of transparency on the part of the government regarding the app’s operation [67].

Additional concerns include a reliance on private tech corporations to provide location data, echoing concerns of public-private partnerships that ring similar to those voiced about the Apple/Google GAEN API [67]. Alarms have been raised regarding the potential for Alipay and WeChat to share information with police agencies [33]. Likewise, this public health role provides large tech companies with unprecedented access to individual health information, which concerns some users due to the overall lack of transparency in this process. It remains unclear how exactly public and private entities manage data collected through the Health Code, who owns this data, and how the government regulates the Health Code [33].

### South Korea

The South Korean contact tracing app, Self-quarantine Safety Protection App, was mandatory for all citizens, and data was stored in a centralized database. Citizens could then view the database and see people’s whereabouts to determine if they were at risk [68]. One critique of this system was that users had to read through a lot of information daily, such that users stopped actively checking the updated information. Another app, Corona 100 m, created a more streamlined service to identify infection hotspots to avoid information fatigue [69]. However, one of the most common critiques of the South Korean system was the lack of privacy, security, and protection from fellow users/laypeople and protection of the business. Protection from identification is essential to avoid stigmatization of any individual or businesses, especially in the worldly context of economic strain due to the pandemic [70]. 

Introna and Poulodi define privacy as the protection from judgment from others, a highly relevant concept when considering the case of privacy breaches from contact tracing apps in South Korea [70-72]. In South Korea, businesses were threatened with false reports on site. Further, reporting of cases in or around the region of businesses resulted in economic strain, boycotting, and riots. This relates to Rowe’s [9] first condition for a contact tracing system’s success: correctness of the information on the app. This first point has two factors to consider: all diagnostic tests have some rate of false negatives and false positives, which may be reduced but not eliminated. Another factor is self- versus physician-reporting of positive cases. If users self-report, security issues arise, including the possibility of false reporting to stir anxiety and fear or even attack a specific location or business, as seen in South Korea. The latter point touches on the issue of privacy for businesses: by sharing the specific location of the infection, businesses around that area are now at risk of financial strain or even boycott. However, if physicians must report positive cases, the app would acquire less information, with those unable to go into a doctor’s office or hospital unaccounted for. Further, whether a business is identified as a hotspot for infection due to blackmail, or it simply was the location of a positive contact point, the business may still be placed at an economic disadvantage. That information about the business is now being shared, potentially without their knowledge or consent, and they may be judged as a result. Methods of contact tracing which use GPS, QR codes, or any location-specific identifiers that are freely shared among users put businesses at risk.

However, businesses were not the only parties affected by South Korea’s system. Personal information about individuals was discovered or speculated, leading to the stigmatization of those individuals. This result was especially damaging due to the fact that participation in South Korea’s system was mandatory, rather than opt-in or opt-out. Therefore, those infected had no means of privacy from widespread stigmatization from their peers. Even without specific name sharing, individuals experienced online attacks, and events involving collective action against individuals online have been reported [73]. After an outbreak in an area associated with gay clubs, many individuals did not get tested or quarantined out of fear that they would out themselves and be judged by their community. It has been suggested that lottery-style randomized testing may protect against issues such as this [74].

### Norway

Norway’s Smittestopp app provides a key example of how rushed development of sensitive technologies can detrimentally backfire. The Smittestopp app used both GPS and Bluetooth, which was stored centrally on a government-controlled cloud platform [75]. The app collected data including mobile phone numbers, age, location data, and contact with infected individuals [75]. The app was said to collect anonymized movement data and would notify users of potential infected close contacts [76]. The app was not open source, which prevented community-based auditing [75]. The hasty development of the app also meant that it had several issues and was not particularly user-friendly, which discouraged its use [75]. Only 1.5 million people (out of 5.3 million) downloaded the app, which was not enough people for useful contact tracing [76]. As such, Norwegian Data Protection Authority deemed Smittestopp illegal because it collected too much personal information without providing a clear data usage policy to its users. The government was advised to shut down the app [76].

Norway’s Smittestopp app was forced to undergo significant restructuring in mid-June by the government since the number of cases could not justify surveillance of the people [76]. Norway’s rushed development of an app resulted in a less-than-optimal program, with significant security risks. Such an example highlights the need to be careful, thoughtful, and deliberate in the creation of such an app, especially when it comes to privacy concerns. Without doing so, privacy breaches may lead to a public that lacks trust in the app. After that point, it may be difficult, if not impossible, to acquire trust again. It is essential that as governments and organizations move forward, these apps are prioritized privacy and security prior to distribution to avoid wasted effort, resources, and time.

Despite its limitations and flaws, the app was at first successfully deployed because Norwegian culture places high trust in the government and novel technologies. What may be learned here is that full trust in novel technologies can lead to security and privacy breaches. However, a full distrust in novel technologies prevents the implementation of innovations that could be beneficial to local and global communities. Therefore, all of these digital contact tracing apps together (summarized in Table 2) show that a healthy skepticism level, as well as standardized and timely auditing, will improve future digital contact tracing technologies

**Table 2:** Summary of key implementation decisions of each described contact tracing app.

App name	Country/company of origin	Centralized/ decentralized/hybrid	GPS/ Bluetooth/ other	Google/ Apple API^a^?	Mandatory/ opt-in/ opt-out/ other
TraceTogether [33,55,56,58]	Singapore	Hybrid, centralized reporting with a confirmed case	Bluetooth	No	Opt-in
Google/Apple Exposure Notification API [45,50,59]	Apple/Google	Decentralized	Bluetooth	Yes	Opt-in
COVID Tracker [62-64]	Ireland	Decentralized	Bluetooth	Yes	Opt-in
Health Code [33,66-68]	China, Alipay/WeChat	Centralized	GPS, symptom tracking; QR^b^ code	No	Mandatory
Self-quarantine Safety Protection App [69-74]	South Korea	Centralized	GPS	No	Mandatory
Smittestopp [75,76]	Norway	Decentralized	Bluetooth	Yes	Opt-in

^a^API: application programming interface.

^b^QR: quick response.

## Primary Limitations and Concerns in App Design

### Privacy and Security

#### Privacy and Security Limitations and Concerns

A high proportion of the local and national population must use a contact tracing app for the app to be successful [9]. This notion is intimately tied with privacy, security, and autonomy. Public perception and acceptance play a key role in app downloads and usage for voluntary opt-in and opt-out systems. However, notions of privacy differ from country to country, and local perceptions will influence which implementation decisions are acceptable. All contact tracing will require some privacy loss, so each community must determine their optimal privacy-utility tradeoff. When public health and wellness are at odds with privacy, this tradeoff may be different than in times of stability. Several surveys have been conducted worldwide to understand opinions about digital contact tracing apps, and the primary cited reasons for not using apps were privacy, security, and surveillance. Given the large concern from users, privacy, security, surveillance, and transparency must be at the forefront of future and current contract tracing app designs.

In Ireland, of those surveyed who reported that they would not download the app, the most common reason was privacy [77]. They found concerns that the app host would use personal data for surveillance purposes, rather than for public health, and that surveillance would continue after the pandemic. Similarly, a survey study in Jordan found that 71.6% agree with the use of contact tracing apps, but only 37.8% used such technology. The main concerns among survey participants were privacy, voluntary status, and beneficence of the data [78].

In a recent large-scale, multicountry study with 5995 participants, the surveyors found general support for contact tracing apps [79]. However, they note that participants from the United States were generally less supportive of the app than other respondents. They found that this generally corresponded with a lack of trust in the national government. Similar to the Ireland study, the main reasons given for not downloading the app were concerns related to government surveillance (42%) and cybersecurity (35%). Interestingly, they found that 74.8% would definitely or probably download an opt-in app, but only 67.7% would probably or definitely keep an opt-out app. Therefore, concerns of privacy, security, and autonomy are primary concerns for users, especially in the United States.

In a Johns Hopkins study of US citizens, 82% reported that they would use a “perfectly accurate and private” tracing app, but only 24% to 26% would want one with even “a low chance” of a data leak to the government, an employer, a tech company, or a nonprofit organization [80]. Given the benchmark that 80% of smartphone users should download a contact tracing app for it to be most successful, these results highlight the essential nature of a deliberately chosen system, which is private and full transparency by entities with an active role in its execution. Without a promise of privacy, it is far less likely that a contact tracing app will be adopted to the degree necessary for efficacy, making it significantly less useful in public health efforts.

Given that even a low chance of data leak is enough to dissuade over 50% of US users, these apps must be reliable and able to maintain a positive reputation. Even a single privacy leak event or controversy will result in a lack of credibility for the app. After such an event, the app would unlikely be viewed as “perfectly accurate and private,” preventing it from reaching the >80% margin necessary for maximal efficacy. Therefore, privacy concerns must be at the forefront of contact tracing app design. Contact tracing apps, especially in the United States, should protect users (both individuals and businesses) from fellow users, the authority hosting the app, and snoopers or hackers.

#### Privacy and Security Recommendations for App Development

In the time of a pandemic, certain rights may be loosened for public health and safety, for example, requirements such as mandated quarantines and mask wearing [81-84]. While disagreement has arisen with these measures, there is a general sense of compromise for the sake of public health [13,85]. When it comes to contact tracing mobile phone apps, a central question is what privacy-utility tradeoff fits within a local community’s values. Will people allow personal location information and contacts to be shared so that the pandemic could be slowed, lives could be saved, and quarantine restrictions could be relaxed sooner? If so, what are the limits of loss of privacy? Inherently, for these apps to function, some degree of location information must be shared. There is an ethical balance between reducing the havoc of the pandemic and saving lives and risking some loss of privacy [68]. Rowe [9] hypothesized that individuals might be comfortable with the risks of location sharing if that meant increased health and financial protection for themselves and their families. Ghose et al [13] found that Americans increased their rates of location sharing services during the pandemic, suggesting that people were comfortable reducing some levels of personal privacy for the sake of public health. Attitudes in South Korea show that the population is comfortable with privacy leakages for the sake of public health [68], whereas reports in American and many European countries favor privacy over public health interventions [79,80].

Nevertheless, in the design of these apps, the likelihood of an information leak should be minimized and not purposefully done. While individuals may sacrifice privacy for the public, unnecessary risks must be minimized. This requires purposeful security design, transparency about data use that should be exclusively for public health purposes, and a promise of nondiscrimination both at the hand of the app host and fellow users. To ensure these requirements, there must be a balance between minimizing information sharing while maximizing the usefulness of the app itself [18]. Yannakourou et al [86] outlined several guidelines. For example, the minimum amount of individual information must be gathered that still allows for efficacious operation, meaning the removal of outdated data that is older than the incubation period of approximately 2 weeks. Unnecessary additional information is deemed unethical since it serves no purpose for public health but is instead collected for the sake of data collection itself. Singapore’s reported 21-day limit follows this guideline. Furthermore, the app should cease use after its benefits are no longer needed (eg, at the end of the pandemic). If these practices were implemented and advertised, this would also further garner trust and, therefore, increase the population’s usage.

While these concerns relate to privacy and transparency from the central host of the app or from fellow users, the app should also be safe from snoopers. For instance, only 16 of 50 apps reviewed prioritized data encryption and security via data anonymization and aggregate online reporting in one study [87]. This concern reinforces the importance of not rushing to deployment but rather carefully confirming the protection of the app from any type of information leakage. Open-source technology and community-based auditing have been suggested as one means of achieving thorough security [80]. Such transparency encourages trust, allows third parties to confirm the intentions of the app host, and allows the technology to be further improved, including cryptographic methods. The Massachusetts Institute of Technology (MIT) Private Kit: Safe Paths has been commended as an open-source, secure, and decentralized program [88]. The MIT implementation was designed with privacy as a priority, and their transparent, open-source model prevents abuse on the part of the app host. Many others have called for increased levels of transparency, whether that be clear and understandable statements regarding the use of data collection or open-source code available for audit [66].

Since privacy-related concerns were the main cited deterrent to app usage, privacy-by-design implementations are necessary for a successful contact tracing app. An app must be thoroughly vetted and trusted for its ability to maintain its users’ privacy and security. An opt-in system may be best in that case, especially in the United States, as trust in government is lower than in other nations [66]. Regardless, this may require trust in the government or in the authority hosting the app through transparency and decentralization. Open-source implementations allow for outside auditing and clear understanding between the developer and the user to increase transparency and accountability. This is a large concern from those who do not wish to download such apps and feel that the government may be using their data for reasons outside of public health and slowing the virus. If the program is trustworthy by open-source practices and transparency and follows proper ethical guidelines, the app may garner more users.

Several other important ethical aspects must be considered when designing such apps, as has been emphasized by several reviews [15,16,18]. It has also been emphasized that mandatory downloading is not an appropriate solution, as not everyone has access to smartphone technology. Similarly, there are concerns about how contact tracing can be used to free people from quarantine in an equitable and fair manner, given that not everyone has access to this technology [15]. Others have brought up concerns about the use of such apps for surveillance and policing of marginalized populations [89]. There are also concerns that centralized servers may risk individual privacy from state surveillance and third-party data breaches. However, centralized information allows for future epidemiology research or public health service planning.

In sum, there are many ethical concerns surrounding contract tracing apps, many of which pertain to privacy, security, and surveillance. Given that a large number of people must actively use contact tracing apps for them to work, opt-in implementations must be trusted by their user base. There is no perfect formula for success in implementation, in large part because each community has its own values and will require a unique approach to the privacy-utility tradeoff. Local laws may also place constraints on what is possible. Surveys of local populations can be used as a springboard to app design. App designers must consider local political and cultural attitudes toward technology, privacy, and public health. Politics can determine regulations on app data collection, and so political norms may dictate the limitations of app functionality. Further, cultural attitudes are likely to influence whether local people are willing to download and use the app at all. Of course, political and cultural attitudes can influence one another. Politics are often a reflection of general cultural expectations. We have highlighted several key contact tracing apps designed specifically based on local political and cultural attitudes, such as the apps in Singapore, South Korea, and Norway. These app implementations may not have been successful or possible at all in countries other than their origin. As such, it is of utmost importance to have a good understanding of local attitudes and needs to ensure that the app is most effective. Future contact tracing apps in the United States will require high levels of privacy protection; open-source and transparent design; and a decentralized, opt-in system.

### Nonprivacy Concerns Related to Apps

As previously discussed, high and consistent long-term usage is essential for contact tracing app efficacy. Although privacy is a key element limiting the extent of usage for this current generation of COVID-19 apps, several nonprivacy factors remain at play in driving users against consistent long-term usage.

#### App Efficacy and Perception Issues

Issues with app performance have resulted in significantly decreased usage. One of the most impactful examples is excessive battery usage due to contact tracing apps. In addition to privacy, Singaporeans have cited depletion of mobile phone battery life as one of the primary reasons against the TraceTogether app [17]. Thus, even in a nation with high levels of digital inclusion and public governance, less than a quarter of Singaporeans downloaded the app initially [55].

Additionally, in response to a software bug in the exposure notifications system developed by Google and Apple [90] caused by the recent update to the GAEN API [91], 83,000 users of Ireland’s COVID Tracker app (out of 1.5 million users) deleted it from their mobile devices in an attempt to solve the underlying technical issue [92]. As a result of the Health Service Executive working with Google and NearForm (the Ireland-based company that designed the app) to solve this Android-specific problem, 10,000 users have already reinstalled the app. However, in a recent October 2020 survey, over half of respondents stated that their app’s Bluetooth technology adversely affects device battery life [78]. These examples highlight the difficulty in regaining users once challenges are encountered and users are lost. Therefore, it is essential that extensive prerelease testing is performed to determine any underlying issues that could affect widespread usage.

While this technical debacle resulted in a loss of 73,000 app users, rapid responses to such issues may mitigate such nonprivacy factor effects on consistent long-term usage. Other than the bug affecting users of the COVID Tracker in Ireland, there have been no other significant reports of battery draining and overheating issues caused by a COVID-19 app. For example, nations such as Canada have seemingly experienced no battery drainage issues, even though they have also utilized the Apple-Google framework [93]. However, even if there were to be future issues, several organizations have resorted to building their own “localized” platforms with no dependence on the GAEN API [94]. For instance, Virginia’s state government reports that they will forgo using the GAEN API for their system, but this may result in the need for significant workarounds for interoperating system communication that results in subpar performance [95]. The lack of transparency and comparability in app design may result in a reduced ability to resolve these issues and decrease app trust in the public.

Another possible factor in limiting the broad usage of COVID-19 apps is a lack of belief in such apps’ effectiveness. One element of this distrust is evident in a survey by the Italian polling organization SWG [96]. In a survey asking 800 individuals, “Why did you choose not to download the Immuni app?,” the most popular reason was “I do not consider it effective” (44%), after which was “I’m afraid for my privacy” (29%).

Finally, a significant factor against the broad usage of contact apps is an emerging sense of complacency. In a report by the Centers for Disease Control and Prevention, 41% of respondents have faced mental health challenges related to COVID-19 and steps taken to combat the pandemic, including social distancing and stay-at-home orders [97]. The literature has also revealed that stressors such as longer quarantine duration, fears of infection, frustration, boredom, inadequate supplies, inadequate information, financial loss, and stigma have contributed to negative psychological effects, including posttraumatic stress symptoms, confusion, and anger [98]. Downloading an app represents another self-sacrifice citizens have to make alongside activities like social distancing and mask wearing that are already mandated or strongly recommended. The long-term nature of the COVID-19 pandemic may lead to complacency when given the opportunity to make another COVID-19–related personal decision.

The literature has revealed that a decrease in concern, in addition to low political trust, can combine to undermine compliance with governmental restrictions during the pandemic [99]. This complacency can significantly adversely affect the adoption of contact tracing apps, even if the ruling government recommended them.

#### App Efficacy and Perception Recommendations

While issues like complacency may stem from the external issues dealing with pandemic fatigue, there are potential solutions for app efficacy and public perception. Bluetooth LE technology, the currently preferred technology for decentralized apps aimed at increasing user privacy, must be demonstrated to consistently and correctly measure user proximity. As of now, Bluetooth LE measurements are subject to discrepancies in signal attenuation based on the specific device used, the relative orientation of devices, and signal absorption and reflection by bodies, handbags, walls, etc [45]. Additional research must be conducted to increase the accuracy and efficacy of the employed technologies if contact tracing apps are to play an impactful role in infectious disease control moving forward. Toward this end, the National Institute of Standards and Technology, along with the MIT PACT (Private Automated Contact Tracing) project, has issued its “too close for too long” challenge to engage with research organizations worldwide concerning noise reduction and more precise distance and temporal estimation of Bluetooth LE signals [100].

Efforts to reduce unwanted false positives and false negatives have evolved in several directions. The Hamagen app used by Israel, for instance, allows users to see the location and time of potential exposure and indicate if they were not present [10]. Alternatively, data from contact tracing apps could be compared with manual tracing efforts to confirm or refute false positives and negatives, and health care providers could be called upon to report and authenticate data to reduce false negatives [10]. Additionally, big data could be used to filter out false positives in a centralized system.

For proximity-based strategies as well as location-based techniques, simulations and modeling may be used to guide design in terms of efficacy and community tailoring. For instance, Pandl et al [101] recently developed and implemented a spatial proximity simulation, which looked at the effects of both proximity detection range (0.2-10 meters) versus contact tracing app adoption (20%-100%), including simulations of decreased use with increase false-positive rates (25%-100% false positives). At higher adoption rates, longer proximity detection ranges (2 meters, 10 meters) were most effective at reducing disease spread but also resulted in increased false positives, which triggered decreased app utilization in highly reactive scenarios. The authors emphasized that this indicates the need to tailor app parameterization and strategies to match cultural expectations, indicating that less sensitive methods involving Bluetooth, GPS, and QR codes would be more acceptable in countries comfortable with a trade-off of high false positives for high efficacy.

In a recent review discussing digital technologies for contact tracing apps, Trivedi and Vasisht [11] discussed research to improve upon existing Bluetooth LE signals such as time-of-flight measurements using Bluetooth that allowed for accuracy to the foot and hybrid techniques such as those employing Bluetooth LE and acoustic-ranging in conjunction, although these have not been widely validated and are not ubiquitously deployable with today’s smartphone technology. Alternatively, merging technical strategies may result in increased overall specificity, particularly for popular Bluetooth-based approaches. A recent paper from Nguyen et al [46] explored the idea of a multi–smartphone-sensor system for contact tracing using Bluetooth combined with barometer (effected by altitude and winds for indoor or outdoor environments), magnetometer (dynamic time warping used to interpret magnetic field vectors), microphone (high frequency, low amplitude short chirps emitted and time of flight measured), and WiFi data (reliant on a grid of WiFi hotspots). With added distance-based readings (microphone, WiFi), accuracy was increased from 25% to 65%, and the additional inclusion of environmental readings (barometer, magnetometer) further increased accuracy to 87%. It should be noted, however, that the significant reduction in false positives was accompanied by a small increase in false negatives. However, these results indicate potential for increased efficacy by the synergistic leverage of multiple sensors for proximity-based digital contact tracing.

Finally, in order to increase public trust in contact tracing app efficacy, apps must undergo extensive testing prior to public release. Moreover, this testing must include rigorous evaluations of app efficacy in real-world environments, particularly those in which users are most likely to have encounters that are difficult to trace, such as public spaces. For example, Leith et al [48] have done testing of the GAEN framework in settings such as public transportation. The efficacy of these apps is not as simple as ensuring customer satisfaction, although this is important for maintaining consistent use, but has become a question of public health as such apps are embraced by national governments worldwide. As such, Bhatia et al [102] suggested that mobile health (mHealth) tools such as contact tracing apps may require regulatory intervention and need the introduction of regulatory sandboxes for extensive beta testing among diverse populations in diverse environments [102]. This strategy would ensure that efficacy data were available and rigorous before the release of a public health app in a time such as our current pandemic, which could both increase faith in the intervention and ensure that it is an appropriate allocation of resources. Appropriate and expedient evaluation of digital contact tracing apps will help determine if the benefit of this technology is worth the potential privacy and security risks previously discussed. Colizza et al [103] have called for such evaluation, and they suggest the use of surveys, epidemiological analysis, and experimental studies. At the time of writing, such analyses were minimal. With the lessons gathered from the use of contact tracing apps for COVID-19, better technology should be available for urgent and efficacious deployment in the event of another infectious disease outbreak. Because contact tracing apps call for some surrender of private data, it is necessary first to ensure that the technology used will be effective in the environments where it is most needed.

To our knowledge, the only evaluation of a digital contact tracing app was reported by Salathé et al [104] on the SwissCovid app in Switzerland, which uses the exposure network framework. They demonstrated a proof of principle that the app reached appropriate contacts who later tested positively for SARS-CoV-2. They provided evidence that notified users subsequently sought SARS-COV-2 testing. This suggests that the SwissCovid appropriately notifies people to self-isolate if they are at risk, which can limit transmission as a result. Nevertheless, there has been model-based analysis on contact tracing in general, which has found that contact tracing can reduce the effective reproduction number of a virus if the tracing is done with minimal delay, the tracing is accurate, and those informed of their potential risk follow appropriate isolation protocols [105]. This research suggests that mobile app technology can reduce the tracing delay, and thus, digital contact tracing apps may minimize viral transmission. Other models have suggested that smartphone-based contact tracing apps can become particularly effective after the first wave of an outbreak. The limitations to effectiveness, as discussed, include the rate of download and use, as well as the accuracy of the app [30]. Still, other than the analysis of the SwissCovid and these theoretical models, evaluations of specific contact tracing apps were not readily available at the time of writing.

## Conclusion

In conclusion, there were many lessons learned from contact tracing apps that were designed to slow the spread of COVID-19. Many of these lessons relate to one core theme: for such an app to work, it is absolutely required that the app be used by a significant portion of the population. For this requirement to be satisfied, the app must offer three main capabilities to its user: (1) a level of trust in the efficacy of the app itself, which requires proper functionality and testing; (2) a level of security and privacy from the app host, fellow users, and malicious entities; and (3) minimal cost or effort from the user, whether that be in the form of battery usage, manual and frequent use of the app, or a host of other factors. Some of these requirements are obligatorily balanced. Many of the apps that proved unsuccessful so far did not sufficiently meet requirement 1 and potentially requirement 2. Therefore, many of these apps have been unable to gain and retain the number of users needed for accurate contact tracing, rendering them ineffective. It was proven repeatedly that these apps must be properly vetted and tested in multiple aspects prior to deployment, as technical performance issues have led to decreased public trust and usage. Likewise, any legal and ethical considerations concerning privacy must be adequately addressed before releasing an app for public use. To avoid the rushed time constraints from an emergency setting, proper development of such apps will need to happen prior to the emergency. In other words, similar to many other issues, we must be proactive rather than reactive when it comes to the use of contact tracing apps moving forward. Likewise, transparency from all actors involved in the development and management of contact tracing apps is necessary. The use of contact tracing apps during the COVID-19 pandemic will improve contact tracing apps in general by providing these real-world lessons.

## Abbreviations

AI: artificial intelligence
API: application programming interface
CCTV: closed-circuit television
CoV-SCR: COVID-19 Symptom Monitoring and Contact Tracking Record
GAEN: Google/Apple Exposure Network
GIS: global information system
mHealth: mobile health
MIT: Massachusetts Institute of Technology
PACT: Private Automated Contact Tracing
PEPP-PT: Pan-European Privacy-Preserving Proximity Tracing
QR: quick response
R0: basic reproduction number
RPI: Rolling Proximity Identifier
STRONG: Spatiotemporal Reporting Over Network and GPS
TTP: trusted third party
